# Muscle Synergies and Coherence Networks Reflect Different Modes of Coordination During Walking

**DOI:** 10.3389/fphys.2020.00751

**Published:** 2020-07-24

**Authors:** Jennifer N. Kerkman, Annike Bekius, Tjeerd W. Boonstra, Andreas Daffertshofer, Nadia Dominici

**Affiliations:** ^1^Department of Human Movement Sciences, Faculty of Behavioural and Movement Sciences, Amsterdam Movement Sciences & Institute for Brain and Behavior Amsterdam, Vrije Universiteit, Amsterdam, Netherlands; ^2^Department of Neuropsychology and Psychopharmacology, Faculty of Psychology and Neuroscience, Maastricht University, Maastricht, Netherlands; ^3^Neuroscience Research Australia, Randwick, NSW, Australia

**Keywords:** interlimb coordination, muscle synergies, muscle networks, locomotion, electromyography

## Abstract

When walking speed is increased, the frequency ratio between the arm and leg swing switches spontaneously from 2:1 to 1:1. We examined whether these switches are accompanied by changes in functional connectivity between multiple muscles. Subjects walked on a treadmill with their arms swinging along their body while kinematics and surface electromyography (EMG) of 26 bilateral muscles across the body were recorded. Walking speed was varied from very slow to normal. We decomposed EMG envelopes and intermuscular coherence spectra using non-negative matrix factorization (NMF), and the resulting modes were combined into multiplex networks and analyzed for their community structure. We found five relevant muscle synergies that significantly differed in activation patterns between 1:1 and 2:1 arm-leg coordination and the transition period between them. The corresponding multiplex network contained a single module indicating pronounced muscle co-activation patterns across the whole body during a gait cycle. NMF of the coherence spectra distinguished three EMG frequency bands: 4–8, 8–22, and 22–60 Hz. The community structure of the multiplex network revealed four modules, which clustered functional and anatomical linked muscles across modes of coordination. Intermuscular coherence at 4–22 Hz between upper and lower body and within the legs was particularly pronounced for 1:1 arm-leg coordination and was diminished when switching between modes of coordination. These findings suggest that the stability of arm-leg coordination is associated with modulations in long-distant neuromuscular connectivity.

## Introduction

Human locomotion requires a well-organized activation of multiple muscles to coordinate movements of upper and lower limbs. The degree of interlimb coordination can be characterized by the strength of frequency and phase locking between limbs. To understand the emergence of coordination patterns and, by this, the way muscle activity is orchestrated, one typically challenges the stability of phase locking by altering a control parameter. For example, if speed is increased from loaf (very slow) to normal walking, one can observe a switch in frequency locking from a 2:1 to a 1:1 ratio between the arm and leg swing (Craik et al., 1976; Schöner et al., 1990; Van Emmerik and Wagenaar, 1992, 1996): At very low speeds, the arm swing is phase locked to the step cycle, while at fast speeds it locks to the stride cycle. This switch is accompanied by a change in the phase relationship between the arms from in-phase to antiphase phase locking (Wagenaar and van Emmerik, 2000), and in the immediate vicinity of the transition the variability of frequency (phase) locking drastically increases^1^. The methodological benefit of investigating such changes in coordination is that they arguably share characteristics of classic phase transitions, in the sense of non-equilibrium thermostatistics (Kelso, 1995; Beek et al., 2002) al., 2002; Kelso, 1995). In the vicinity of a phase transition, one may expect the dynamics’ dimensionality to be drastically reduced and muscle activity patterns to stay on low-dimensional manifolds.

Interestingly, the switch in coordination during walking depends on whether the walking speed is increased or decreased (Schöner et al., 1990; Van Emmerik and Wagenaar, 1996). This suggests that the underlying mechanisms are not purely mechanical or energetic, as has been conjectured in other cases of altered interlimb coordination (Hoyt and Taylor, 1981; Owaki and Ishiguro, 2017). Our working hypothesis is that the central nervous system substantially contributes to the stability of coordination patterns. As such, we sought to identify (low-dimensional) neural contributions to transitions in upper and lower limb coordination. Well-designed mechanical manipulations may already hint at the relevance and location of such neural contributions. For instance, Bondi et al. (2017) reported how changes of swing of one arm can affect both the swing of the other arm as well as lower limb coordination during walking. The same effects have also been shown in neonates (La Scaleia et al., 2018), children with hemiplegic cerebral palsy (Meyns et al., 2012), and are known for long for stroke survivors where they can be strongly elevated (Stephenson et al., 2009). By the same token, the arm swing can have little to no influence on leg movement after spinal cord injury (Tester et al., 2012). These findings suggest that a partial interruption of the spinal cord may suffice to limit the interaction between spinal motor neurons such that switches in interlimb coordination no longer emerge.

Targeting neural dynamics more directly during motor coordination is not new (Matsuyama et al., 2004). Several groups studied modulations of muscle activity of upper and lower extremities during locomotor tasks via electromyography (EMG) – a proxy of neural activity in the spinal cord (Ferris et al., 2006; Boonstra et al., 2016; Zehr et al., 2016). Muscle activity of different muscles is found to couple at several time or frequency scales. Coherence at low frequencies (0–5 Hz) seems associated with common modulation of motor unit mean firing rate and muscle force generation and, hence, likely reflects co-modulation of muscle activities (De Luca and Erim, 1994; Mochizuki et al., 2006; Boonstra et al., 2008) and the modulation of EMG envelopes (Hansen et al., 2001). Common modulations of EMG envelopes of groups of muscles are considered as muscle synergies (Tresch et al., 2006) that reveal how movements are manifested through synchronized muscle co-activation (Ivanenko et al., 2004, 2005; Cheung et al., 2005; Cappellini and Ivanenko, 2006; Dominici et al., 2011). In a recent review, Bruton and O’Dwyer (2018) outlined numerous studies suggesting that muscle synergies are vital motor control modules. Obviously, muscle synergies change with altered coordination, but what are the origins of these changes? An answer to this may lie in the higher frequencies of the EMG signal, as they may provide the spectral “fingerprints” of distinct neural pathways involved in the control of muscles (Farmer, 1998; Boonstra et al., 2009a, 2016; Danna-Dos-Santos et al., 2014). For example, intermuscular coherence at higher frequency components may reflect supra-spinal drives (Grosse et al., 2002) that modulate the activation of multiple muscles by means of a common input (Danna-Dos-Santos et al., 2014).

Here, we studied the dynamics of muscle activation during changes in interlimb coordination using the experimental design of Wagenaar and van Emmerik (2000). Rather than focusing on isolated muscles, we employed synergy analysis and constructed functional muscle networks (Boonstra et al., 2015). We determined the minimal (i.e., low-dimensional) set of muscle synergies and combined them into a network with multiple synergy-specific layers. In a similar spirit, we used intermuscular coherences to construct networks with multiple frequency-specific layers (Kerkman et al., 2018). Both types of networks were constructed under the proviso that they could be based on a low-dimensional representation^2^, i.e., a small number of relevant muscle synergies vis-à-vis a small number of frequency components with pronounced coherence determined through conventional mode decomposition of multivariate time series. Network analysis offers new possibilities to assess synchronization between motor units across a large number of muscles. It hence allows for an encompassing study of functional changes in muscle activity during a transition in physiological coupling (Bashan et al., 2012; Bartsch and Ivanov, 2014). In particular, modulations of the network can highlight modifications in the neuromuscular system related to changes in functional behavior during walking.

For the individual synergies, we expected the switch in interlimb coordination to be accompanied by rapid changes in temporal activation patterns, in line with Yokoyama et al. (2016). For the corresponding low-frequency muscle networks, we expected a strong resemblance of anatomical and biomechanical constraints (Kutch and Valero-Cuevas, 2012; Bruton and O’Dwyer, 2018) and switches in coordination to result in concomitant changes in network topology. Given that the higher EMG frequency components are thought to represent supra-spinal input to multiple muscles (Kerkman et al., 2018), we expected these frequency components to discern neural pathways involved in the stability of arm-leg coordination patterns and the switches between them.

## Materials and Methods

### Subjects

Sixteen healthy subjects (five males and eleven females, mean age of 25.3 ± 2.4 years) without any neurological or motor disorder were included in this study. The study was approved by the Ethics Committee Human Movement Sciences of the Vrije Universiteit Amsterdam (VCWE-2017-132). All subjects were informed about the procedure of the study and provided, in accordance with the Declaration of Helsinki, written informed consent prior to participation.

### Procedure

Subjects were instructed to walk on a treadmill (Motek Medical B.V., Amsterdam, Netherlands) with their arms swinging along their body while full-body kinematics, ground reaction forces and muscle activities were recorded. Subjects walked at controlled speeds between 1.0 and 4.0 km/h with increments of 0.5 km/h. The ordering of speeds was randomized between subjects and trials. Subjects walked for at least fifteen strides at each speed; see movie ExperimentalParadigm.mp4 in the Supplementary Material.

### Data Acquisition

Ground reactions forces (Motek Medical B.V., Amsterdam, Netherlands) and full-body 3D-kinematics (Optotrak, Northern Digital, Waterloo, ON, Canada), using five cluster markers (heel, lower and upper leg, and upper and lower arm) and three cameras (left and right backside and one at the front), were measured to define the fifth metatarsophalangeal joint, heel, ankle, knee, hip trochanter, shoulder, elbow and wrist. Kinetic and kinematic data were sampled at 70 Hz. Surface EMG of 26 bilateral muscles (Table 1) distributed across the body was recorded (two Mini Wave Wireless 16-channel EMG system, Cometa s.r.l, Italy) and sampled at 2 kHz after online band-pass filtering between 10 and 500 Hz. Electrodes were placed according to the SENIAM recommendations (Hermens et al., 1999). Kinematic, ground reaction force and EMG data were synchronized online.

**TABLE 1:** Muscles included in the recordings.

**Muscle**	**Abbreviation**
1. Tibialis anterior	TA
2. Gastrocnemius medialis	GM
3. Tensor fascia latae	TFL
4. Rectus femoris	RF
5. Vastus medialis	VM
6. Adductor longus	AL
7. Biceps femoris	BF
8. Gluteus maximus	GMA
9. Erector spinae	ES
10. Latissimus dorsi	LD
11. Trapezius	TZ
12. Deltoid	D
13. Triceps brachii	TRB

### Data Analysis

#### Kinematics

**FIGURE 1:**
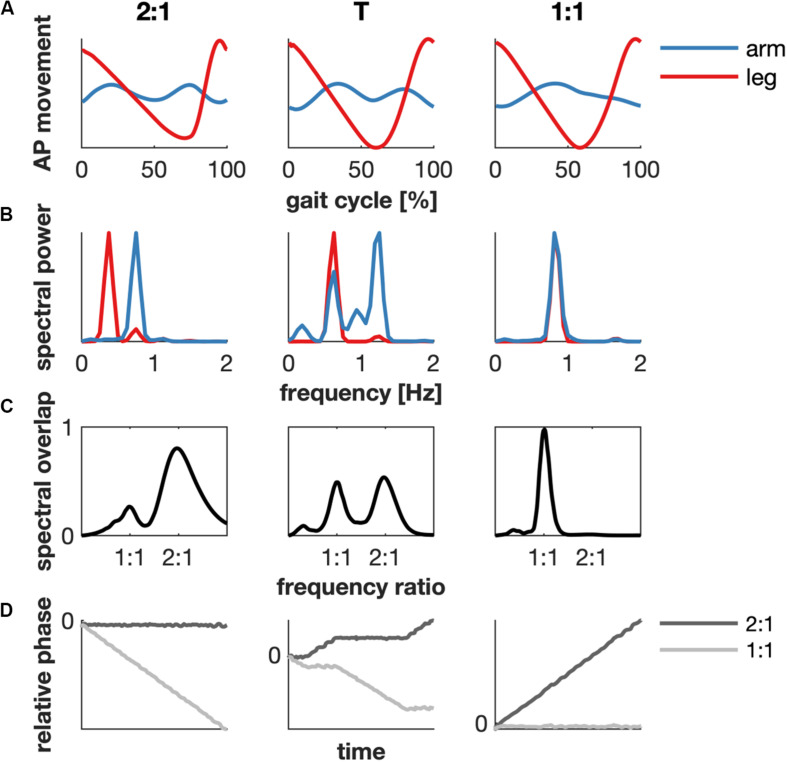
Example of the determination of the modes of coordination based on kinematics of subject 1. **(A)** The average arm (blue) and ipsilateral leg (red) movement in the anterior-posterior (AP) direction as a function of the gait cycle in the 2:1 (1.0 km/h), transition (T, 2.5 km/h) and 1:1 (4.0 km/h) mode of coordination. **(B)** The spectral power. **(C)** The spectral overlap between the power spectra of the arm and leg is maximal for a 2:1 or 1:1 coupling between the arm and leg movement in the 2:1 and 1:1 mode of coordination, respectively. The transition contained peaks at both 2:1 and 1:1 coupling. **(D)** The relative phase between arm and leg. A generalized relative phase of zero slope implies that arm and leg move at a fixed frequency ratio (2:1, black and 1:1, gray).

Gait cycles were defined based on the right heel strikes obtained from the force plate data. The heel strike was defined as the moment when the vertical ground reaction force exceeded 8% of the average ground reaction force during the trial. This kinetic criterion was verified by comparison with foot strike measured from the kinematic data (Borghese et al., 1996; Roerdink et al., 2008). We determined the mode of interlimb coordination via the maximum spectral overlap after rescaling the frequency axis (Daffertshofer et al., 2000) and the circular variance of the generalized relative phase of the kinematics of the arms and legs for every walking speed and subject (cf. Table 2). We focussed on the frequency locking between arms and legs at 2:1 (∼ very low speed) and 1:1 (∼ normal), and the transition (T) between these modes of coordination. The 2:1 and the 1:1 condition were dominated by spectral overlap at a 2:1 or 1:1 frequency ratio, respectively, and almost constant corresponding generalized relative phases. The transition was characterized by spectral overlap at both frequency ratios of 2:1 and 1:1, and a changing generalized relative phase (Figure 1).

**TABLE 2:** Overview of modes of coordination per subject per walking speed.

**Speed\Subject**	**1**	**2**	**3**	**4**	**5**	**6**	**7**	**8**	**9**	**10**	**11**	**12**	**13**	**14**	**15**	**16**
1.0 km/h	2:1	T	T	2:1	2:1	2:1	T	2:1	2:1	T	T	N/A	2:1	T	2:1	T
1.5 km/h	2:1	T	1:1	T	T	1:1	1:1	T	1:1	1:1	2:1	T	T	1:1	2:1	T
2.0 km/h	2:1	1:1	1:1	T	1:1	1:1	1:1	1:1	1:1	1:1	1:1	1:1	T	1:1	T	1:1
2.5 km/h	T	1:1	1:1	1:1	1:1	1:1	1:1	1:1	1:1	1:1	1:1	1:1	1:1	1:1	1:1	1:1
3.0 km/h	1:1	1:1	1:1	1:1	1:1	1:1	1:1	1:1	1:1	1:1	1:1	1:1	1:1	1:1	1:1	1:1
3.5 km/h	1:1	1:1	1:1	1:1	1:1	1:1	1:1	1:1	1:1	1:1	1:1	1:1	1:1	1:1	1:1	1:1
4.0 km/h	1:1	1:1	1:1	1:1	1:1	1:1	1:1	1:1	1:1	1:1	1:1	1:1	1:1	1:1	1:1	1:1

*2:1 represents a double arm swing of both arms during one gait cycle, 1:1 represents a 1:1 coordination pattern with one arm swing of both arms during one gait cycle, and T represents the transition between the 2:1 and 1:1 mode of coordination in which both patterns were observed.*

#### EMG Pre-processing

Independent component analysis was used to reduce heart beat contamination in the EMG signals (Willigenburg et al., 2012). Subsequently, EMG signals were high-pass filtered (2nd order, bi-directional Butterworth, cut-off at 30 Hz) and rectified using the modulus of the analytic signal. Here we would like to note that rectification can re-introduce low-frequency amplitude modulations (Myers et al., 2003; Boonstra and Breakspear, 2012).

#### Muscle Synergies

Electromyography envelopes were determined by low-pass filtering the rectified EMGs (2nd order, bi-directional Butterworth filter, cut-off at 10 Hz). Subsequently, these envelopes were time normalized such that every stride had an equal number of samples (*N* = 200 samples). For every subject we further normalized the amplitudes to the average activity during the fastest walking speed (4.0 km/h)^3^. Next, EMG data for every subject were averaged over all strides per mode of coordination yielding *EMGs* × *subjects* × *conditions* time series containing one average stride each. Finally, time series were concatenated along subjects and conditions yielding 26 (number of muscles) discrete time series containing *subjects* × *conditions* (*SC*) strides each^4^. We denote the data by *X*_ij_ where *i* indexes the time point and *j* the muscle, that is, *i* = 1, …, *SC*⋅*N* spans the *SC* time-normalized strides with *N* samples each and *j* = 1, …,26 are all muscles. These data entered our synergy analysis, namely non-negative matrix factorization (NMF). NMF is a linear mode decomposition *X*↦*W*^(*m*)^*A*^(*m*)^ that includes the constraint that both extracted wave forms *A*^(m)^ and weights *W*^(m)^ are positive semi-definite, and that *W*^(m)^ and *A*^(m)^ have rank *m*; we used a multiplicative update algorithm to solve the corresponding minimization of the Frobenius norm ${||{\text{X–W}}^{\left(m\right)}\,A^{\left(m\right)}||}_{F}^{2}$ (Lee and Seung, 1999).

To fix the number of relevant synergies, i.e., the rank *m* of *W*^(m)^, we determined the quality of data reconstruction as

(1) $$\lambda^{\left(m\right)}=\left(1-\frac{{||{\text{X–W}}^{\left(m\right)}\,A^{\left(m\right)}||}_{F}^{2}}{{||X||}_{F}^{2}}\right)\times 100\%$$

and required λ^(m)^ ≥ λ_cutoff_ = 80% (Zandvoort et al., 2019) and, additionally, λ^(m)^−λ^(^*^m–1^*^)^ ≥ Δλ_cutoff_ = 1.5%. This notion let us also define the contribution of every synergy to the representation of *W*^(m)^*A*^(m)^ by realizing that *W*^(m)^ = [w_1_,…,*w*_m_] and *A*^(m)^ = [*a*_1_,…,*a*_m_]. That is, the contribution of an individual synergy *s* could be given as

(2) $$\lambda_{s}=\left(1-\frac{{||{\text{X–w}}_{s}\,a_{s}||}_{F}^{2}}{{||X||}_{F}^{2}}\right)\times 100\%$$

Note that by combining the signals as described above, we obtained different wave forms between and common muscle weights across conditions and subjects, i.e., fixed muscle groups over conditions with varying activation patterns. For the sake of legibility, in the following we denote these outcomes as *X*↦*W*^(*syn*)^*A*^(*syn*)^.

#### Intermuscular Coherence

The rectified EMGs were down-sampled to 256 Hz to reduce computational load. Data of the same condition were mean-centered and concatenated. Intermuscular coherence was determined between all 26 × 25/2 =325 muscle pairs per subject and condition. The power spectral densities *P*_x_ and *P*_y_ of signal pairs (*x,y*) and the complex-valued cross-power spectral density *P*_xy_ were estimated using Welch’s periodogram method (Hamming taper of 200 ms length and about 50% overlap). With this we computed the squared coherence $C_{x\,y}^{2}=\left(P_{x\,y}\,P_{x\,y}^{*}\right)\,/\,\left(P_{x\,x}\,P_{y\,y}\right)$; here (.)^∗^denotes the conjugate complex.

We corrected the coherence estimates for the bias due to differences in data length. We employed a bootstrapping approach (100 surrogates) of the complex-valued cross-spectral density through phase randomization (Hurtado et al., 2004; Kantz and Schreiber, 2004). In brief, phase randomization destroys coherence implying that the resulting bootstrap distribution is zero-centered. However, due to finite-size estimation the distribution may have a finite, frequency-dependent variance even for infinitely many surrogates. This variance yields a null distribution indicating the absence of coherence, which served as normalization factor for the coherence estimates. Since the latter is the modulus of the normalized cross-spectral density, the resulting distribution of squared coherences is a Chi-squared distribution with two degrees of freedom for which we considered squared coherences below α = 0.05 not distinguishable from chance. Accordingly, these values were set to zero.

In line with the synergy analysis, we concatenated the data, i.e., now the corrected coherence spectra across the frequencies (*f*, 4–60 Hz), over subjects and conditions (SC) and 325 muscle pairs. This yielded a *f* × (SC × 325) matrix, and we applied NMF to obtain *C*↦*W*^(*coh*)^*A*^(*coh*)^. This NMF yielded *m* modes, *W*^(coh)^ = [*w*_1_,…,*w*_*m*_] with *w*_j__=__ 1,__…_,*_m_*, containing *SC* × 325 coherence weights each, and *A*^(coh)^ = [*a*_1_,…*a*_m_], with *a*_j__=__ 1,__…_,*_m_* defining the *m* modes for all subjects, conditions and muscle pairs. To anticipate, these modes separated distinct frequency ranges. From hereon we therefore refer to these modes as frequency components. The number of these components was fixed using Eq. (1) with adjusted cut-off values: λ_cutoff_ = 55% and Δλ_cutoff_ = 4%.

#### Muscle Networks

We constructed muscle synergy and coherence networks with muscles as nodes and their functional connectivity as edges between them. The synergy-NMF yielded *w*_j__=__ 1,__…_,*_m_* that contained 26 muscle activity weights each for every synergy. We used the outer product *W*_j_⋅*W*_j_ to define the connectivity matrix $C_{j}^{\left(s\,y\,n\right)}$ of synergies *j* = 1,…,*m* to create a one mode projection of a bipartite network (Murphy et al., 2018) with *m* layers (Horvát and Zweig, 2012). In this synergy network, every element of the connectivity matrices represented the weighted appearance of two muscles in the same synergy. To include the contribution of the synergies by means of the amplitude of the wave forms, the connectivity matrices were weighted for the sum of the integrals of the wave forms of the three modes of coordination.

The intermuscular coherence weights of the *m* frequency modes (NMF modes) served to define the edges of the coherence network. We thus obtained *m* × *SC* different 26 × 26 connectivity matrices $C_{j}^{\left(\text{coh}\right)}$that we averaged over subjects and combined into an *m* × 3-conditions multiplex network. The community structures across layers of both the synergy and coherence networks were determined by the Louvain algorithm (Jeub et al., 2019).

To compare topological characteristics of the coherence networks between modes of coordination, we determined the global connectivity, clustering of muscles and strength of connections in the network by means of global efficiency, transitivity, and average strength across nodes (Bullmore and Sporns, 2009; Rubinov and Sporns, 2010) for all layers. Before doing so, the corrected coherence networks were thresholded to construct a minimally-connected network across the layers of the network, i.e., every node (muscle) was connected to at least one other node in one of the layers and the number of edges within the layers was constant across the layers.

Additionally, we time normalized the EMG data and estimated coherence again, but now with a Hamming taper of 5 s over the 0.6–4 Hz frequency range to directly compare synergy and (very) low-frequency coherence networks. Details of this analysis can be found as Supplementary Material.

#### Statistics

Statistical differences between conditions were assessed over subjects who exhibited both conditions (either 2:1 and transition, 2:1 and 1:1, or transition and 1:1).

Changes in the synergy wave forms were compared in two ways. First, we compared the amplitude during the gait cycle between modes of coordination. Subsequently, the amplitudes were normalized to the maximum of the wave form and we compared the amplitude-normalized wave forms between modes of coordination. We determined the samples of the time series which were significantly different in either amplitude or wave form between the conditions using statistical parametric mapping including paired *t*-tests (Pataky et al., 2015; see also www.spm1d.org). Significance was identified based on an alpha threshold value corrected for multiple comparisons in three conditions and five synergies, i.e., α = 0.05/(3.5) = 1/300.

Differences between the network metrics of the layers of the coherence networks, i.e., modes of coordination and frequency components, were compared with a univariate ANOVA with subject as random factor (α = 0.05). *Post-hoc* tests were performed to examine differences between conditions per frequency component (α = 0.005).

## Results

### Behavior

The kinematic assessment of the modes of coordination revealed that only seven subjects showed both modes of coordination and the transition between the two. The 2:1, transition, and the 1:1 mode of coordination appeared in nine, fourteen, and sixteen subjects, respectively (Table 2).

Figure 1 represents a typical example (subject 1) of the movement of the right arm and ipsilateral leg in the sagittal plane, the corresponding spectral power and overlap, and the relative phase for the 2:1, transition and 1:1 condition.

### Muscle Activity

Differences between modes of coordination were clearly visible in both the amplitudes and wave forms of the EMG envelopes (Supplementary Figure S1). EMG amplitudes particularly differed around the heel strike event in the ipsilateral leg and contralateral back and arm muscles in the 1:1 mode of coordination. The peak activity in the arm muscles around the contralateral heel strike shifted to earlier in the gait cycle when the coordination pattern switched toward a 1:1 mode of coordination between arms and legs.

### Muscle Synergies

**FIGURE 2:**
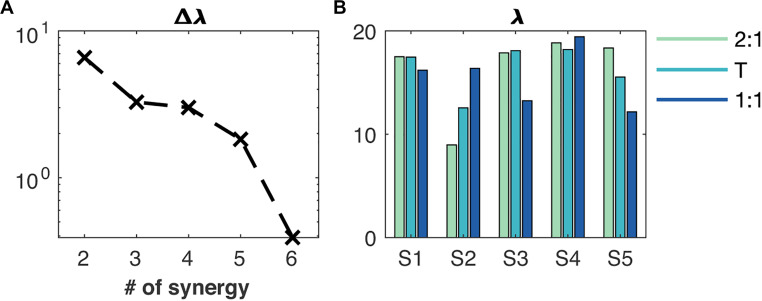
Reconstruction quality of the muscle synergies. **(A)** Additional value of an extra synergy (Δλ) to the total contribution of the synergies to the Frobenius norm, **(B)** the contribution of every synergy (S1 to S5) to the Frobenius norm (λ). The order of synergies S1 to S5 is showed in Figure 3. Green, cyan and blue bar plots represent the 2:1, transition (T) and 1:1 mode of coordination, respectively.

Five muscle synergies accumulated 80% to the Frobenius norm of the original concatenated EMG envelopes and a sixth synergy added very little, which let us fix *m*(syn) = rank[*W*^(syn)^] = 5 (Figure 2). We found $\lambda_{1,\ldots ,5}^{\left(\text{syn}\right)}=\left[17,13,16,19,15\right]\%$ on average across conditions.

**FIGURE 3:**
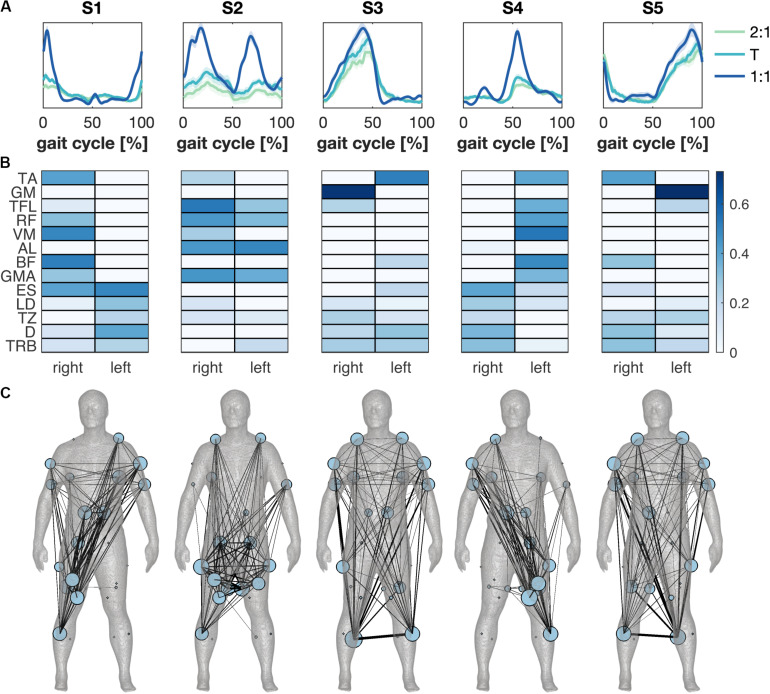
Muscle synergies across modes of coordination. **(A)** The synergies’ temporal activation patterns as a function of the gait cycle derived from average muscle activity patterns for the different modes of coordination. Green, cyan and blue represent the 2:1, transition (T) and 1:1 mode of coordination. Error patches represent the standard error of the mean across subjects. **(B)** Synergies’ weightings across conditions and subjects in color scale. **(C)** Muscle synergy network plotted separately for each synergy on the body mesh (Makarov et al., 2015). A minimally-connected network was created for visualization. Node size represents the degree of the muscle and edge thickness represents weighted appearance of both muscles in the synergy.

Synergies were ordered based on the relative timing of the main peak in the activation patterns (Figure 3A). S1 and S4 were active during the heel strike and weight acceptance response of the right and left leg, while S3 and S5 were active mainly in the calf muscle during the stance phase of the right and left leg, respectively. The muscle weights of S1 and S4 showed activity in both the leg and the contralateral trunk and arm muscles; bilateral calf and contralateral shank muscles were dominant in S3 and S5. S2 was active during the stance and swing phases with primarily activity of muscles around the pelvis (Figure 3B). The contribution $\lambda_{2}^{\left(\mathrm{syn}\right)}$ of S2 increased from 2:1 to 1:1, while $\lambda_{3}^{\left(\mathrm{syn}\right)}$ and $\lambda_{5}^{\left(\mathrm{syn}\right)}$ decreased.

**FIGURE 4:**
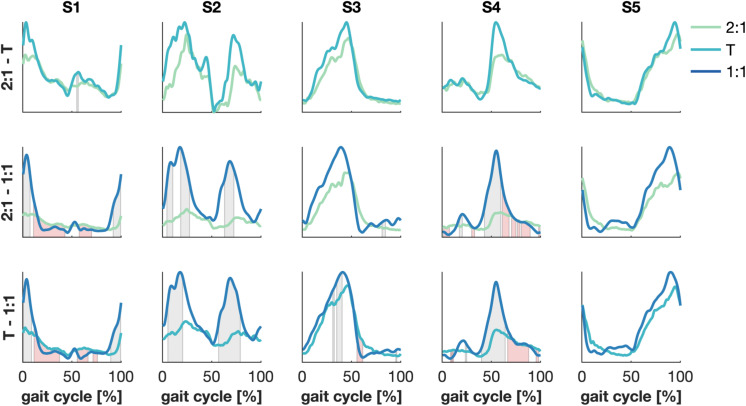
Significant differences between synergies’ wave forms between modes of coordination. Green, cyan and blue represent 2:1, transition (T) and 1:1, respectively. Patches represent significant differences in time between the amplitude (gray) and the temporal patterns (red) of the synergies’ wave forms. α = 1/300.

Significant differences were found between the synergies’ wave forms between the 2:1 and the 1:1 and between the transition and the 1:1 mode of coordination (Figure 4). The amplitude of S1 increased in 1:1 compared to 2:1 and the transition around the right heel strike and the activity decreased quicker with an increase in walking speed. Similar results were found for S4 at the corresponding left heel strike. Changes in the amplitude were also visible in S2 between 2:1 and 1:1 and between the transition and 1:1 during the stance and swing phases of both legs. The activation pattern of S3 revealed some minor differences between the transition and 1:1 in the amplitude halfway the stance phase of the right leg and after the left heel strike, while no significant changes were found for S5.

### Intermuscular Coherence

The coherence spectra were decomposed in three modes, i.e., *m*^(coh)^ = rank[*W*^(coh)^] = 3. These modes reflected distinct frequency bands, 4–8, 8–22, and 22–60 Hz, in line with our previous findings (Boonstra et al., 2015; Kerkman et al., 2018). The frequency components contained in total 57% of the Frobenius norm of the coherence spectra; $\lambda_{1,\ldots ,3}^{\left(\text{coh}\right)}=\left[19,19,19\right]\%$.

We extracted two frequency components (λ_cutoff_ = 19%) from the low-frequency coherence (0.6–4 Hz) showing peaks at 1.5 or 2.5, and 3.5 Hz; $\lambda_{1,2}^{\left(\text{coh}\right)}=\left[9,10\right]\%$.

### Muscle Networks

**FIGURE 5:**
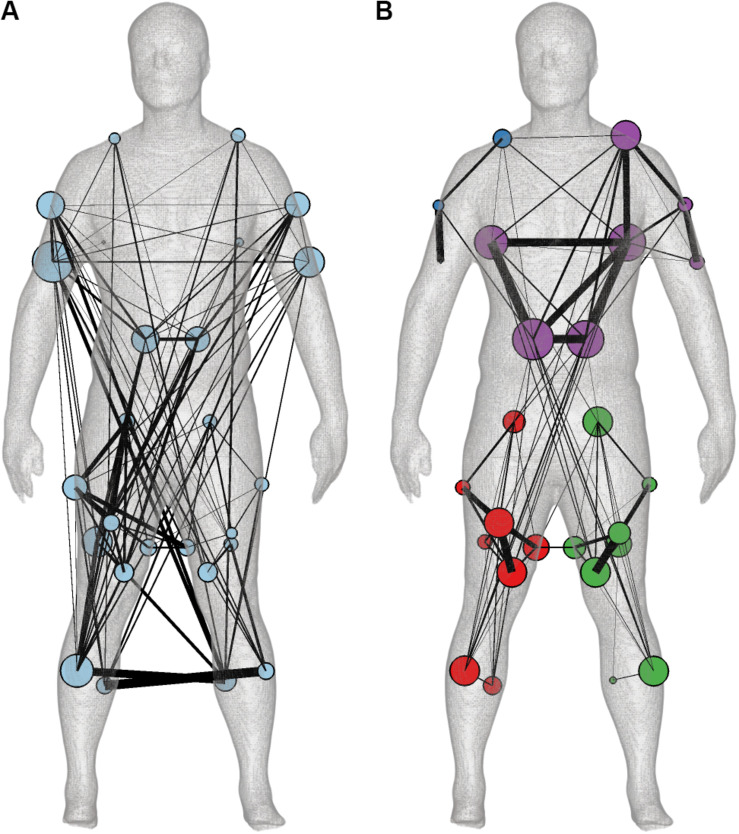
The community structure of the multiplex **(A)** muscle synergy **(B)** and coherence networks based on the synergy and coherence spectra muscle weightings. Community structure is visualized by color-coded nodes and the average degree across layers of every muscle is displayed as node size on the body mesh (Makarov et al., 2015). The edge width is based on the average connectivity across layers between the muscles in either the minimally-connected synergy or coherence network.

Both the muscle synergies and coherence spectra were represented as multiplex networks to facilitate quantitative comparison. For the muscle synergies, each synergy was represented as a layer of the multiplex network (Figure 3C). We subsequently estimated the community structure across all five layers (Figure 5A). As the connectivity in the layers of the synergy network did not overlap substantially, the community structure across layers yielded a single module and the synergy network contained several contralateral connections between arms and legs. These long-distance edges were distinctive for the layers of the synergies active around heel strike (S1 and S4). S3 and S5 also showed symmetries between left and right, but represented a more comprehensive network in which the whole human body was involved. S2 mainly showed connectivity around the pelvis and between the pelvis and the shoulder muscles (trapezius, Figure 3C).

In contrast, the community structure of the multiplex coherence network divided the body in modules of both legs separate, the trunk with the left arm and the right arm (Figure 5B). The average modularity per frequency component was 0.14, 0.30, and 0.32, respectively. By constructing minimally-connected multiplex networks, we removed on average 293 significant edges (threshold was 0.0970) with weights of 0.0015 ± 0.0011 (mean ± standard deviation), 0.0018 ± 0.0011 and 0.0055 ± 0.0039 for 2:1, transition and 1:1, respectively. The preserved edges had weights of 0.0114 ± 0.0077, 0.0114 ± 0.0067, and 0.0184 ± 0.0069. In contrast to the synergy network, the community structure of the coherence network was not affected by this thresholding (see Supplementary Material).

The community structure of the coherence network over 0.6–4 Hz was very similar to the community structure of the coherence network over the frequency range of 4–60 Hz: the Rand and adjusted Rand indices were 0.85 and 0.63, *p* < 0.001, respectively. Yet, individual layers of the coherence network revealed similarities with the layers of the synergy network; cf. Supplementary Material for more details.

#### Changes in Coherence Networks

**FIGURE 6:**
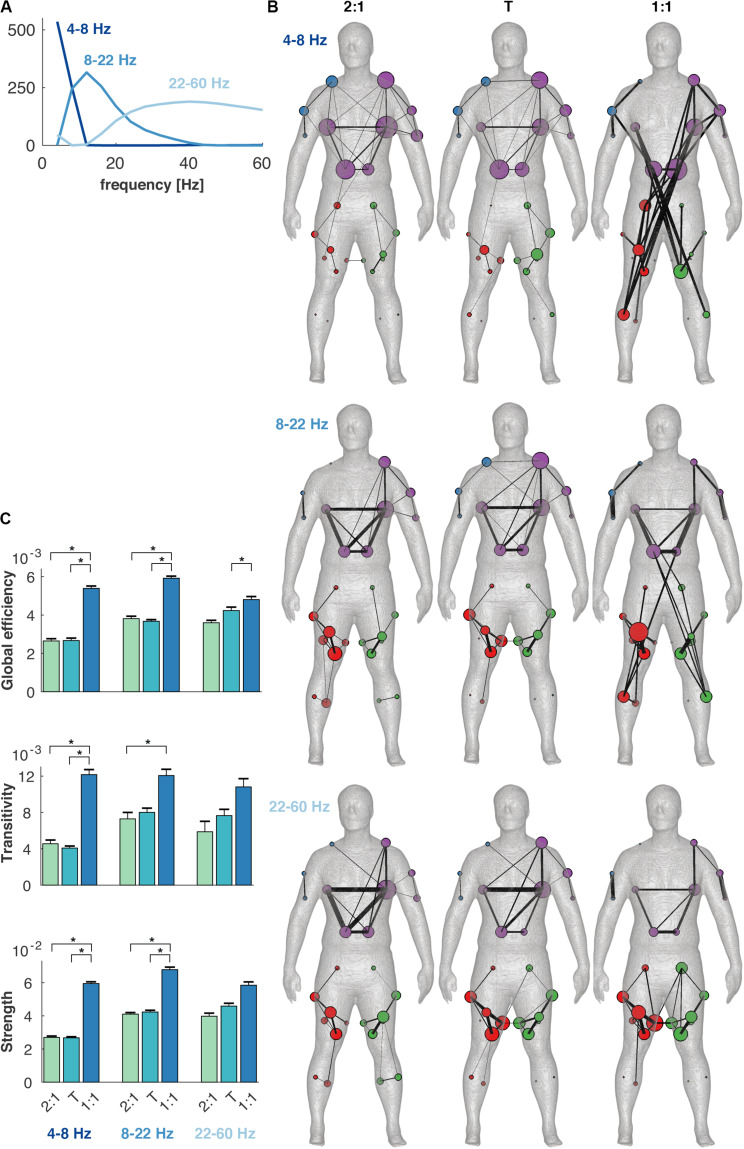
Changes in connectivity between conditions and frequency components in the minimally-connected multiplex coherence network. **(A)** Frequency components 4–8, 8–22, and 22–60 Hz, obtained with non-negative matrix factorization. **(B)** Coherence networks in the 2:1, transition (T) and 1:1 mode of coordination (columns) and the frequency components (rows). Colors in the networks depict different modules and node size and edge width represent degree and connectivity strength between muscles, respectively. **(C)** Global efficiency, transitivity and average strength of the coherence networks per frequency component and condition. Error bars indicate standard errors of the mean and asterisks significant differences between conditions (α < 0.005).

The topology of the coherence network was reorganized when the coordination pattern changed to the 1:1 mode of coordination (Figure 6). The network metrics, i.e., global efficiency, transitivity and average strength, were significantly different between conditions [*F*(2,21) = 56.0, *F*(2,21) = 12.1, and *F*(2,21) = 38.7, respectively, *p* < 0.001]. The 1:1 mode in the 4–8 Hz frequency component contained several long-distance connections between the leg and the contralateral arm with high connection strengths corresponding to a high global efficiency (Figure 6C). In contrast, both the 2:1 and the transition showed mainly connections within and between upper body and arms. At 8–22 Hz, 1:1 coordination again deviated from 2:1 and the transition, and was associated with a relatively high global efficiency, transitivity and strength. Some long-distance connections were found in 1:1 between the legs and the lower back, and high within-module connectivity appeared within the legs. For the 22–60 Hz frequency component, the connectivity was high within the trunk in 2:1 and the transition, while this connectivity was lower in 1:1. In the latter condition, the connectivity was higher between arm muscles. The highest frequency component was without connections between the upper and lower body in all conditions.

## Discussion

The aim of this study was to identify neural correlates of spontaneous switches in interlimb coordination during walking, i.e., transitions in frequency locking ratios between the arms and legs when walking speed changes. We applied more conventional synergy analysis and extended this to multiplex networks in line with the more recently introduced coherence-based muscle networks (Kerkman et al., 2018). As expected, we found changes between task conditions in the activation patterns of specific muscle synergies and in the network metrics of specific frequency layers of the coherence networks. In particular, we found increased activation of the synergies active around right and left heel strike (S1 and S4, respectively) during 1:1 phase locking compared to the other two coordination modes. Likewise, synergy S2 involved the muscles around the pelvis and also showed increased activation during 1:1 locking; note that this synergy appeared left/right symmetric. In contrast, synergies S3 and S5, involved in the initiation of the swing of the left and right leg, respectively, remained largely unchanged across modes of coordination. Similar to the muscle synergies, 1:1 coordination revealed increased connectivity between upper and lower limbs in two (lower) frequency components (4–8 and 8–22 Hz) compared to the other two modes of coordination. The increase in long-distance connectivity was associated with a corresponding increase in global efficiency, transitivity and average strength. We found four modules grouping either left and right leg muscles or left and right arm muscles, though, the module containing the left arm also included all the recorded trunk muscles. These findings indicate that the transition to a 1:1 coordination pattern is associated with a reorganization in the muscle activation patterns.

Arm-leg coordination switched from 2:1 to 1:1 frequency locking mode when walking speed was increased. During the transition period both coordination patterns could be observed supporting the notion of multi-stability (Van Emmerik and Wagenaar, 1996). However, this was not observed in all subjects, in line with earlier studies reporting that the incidence of the 2:1 coordination pattern is reduced in treadmill compared to over-ground walking (Carpinella et al., 2010). Future studies may focus on even lower treadmill speeds to pinpoint neurophysiological changes possibly underlying the transition in coordination. Yet, we identified statistically significant differences between the coordination modes in individual muscle activation patterns. We are confident that these findings underwrite earlier documented importance of arm muscle activity during walking (Craik et al., 1976; Meyns et al., 2013; Goudriaan et al., 2014). They also revealed phase-specific modulations of arm muscle activity associated with the kinematic switches in interlimb coordination (see Supplementary Material). Last but not least, the modulations of EMG activity were reflected in the reorganization of the muscle synergies.

Speed-induced adaptations in muscle synergy strength and timing have been reported earlier (Ivanenko et al., 2004; Yokoyama et al., 2016), which led Den Otter et al. (2004) to speculate that modulations of muscle synergies are a mere by-product of a change in stance and swing time. We found that the synergy active during the stance and swing phases (S2) became stronger accounting for an increase in upper leg activity which may serve to control the relative movement between the trunk and the legs when walking faster. We found left/right-mirrored synergies for both S1 and S4 and S3 and S5; the muscles in S3 and S5 appeared important in preserving the upright body position, while synergies S1 and S4 induced the forward propulsion of the body. Synergies that were active during heel strike were also affected in both the strength and the wave form when switching to another mode of coordination, which was in accordance with the changes in relative timing of the arm swing. The synergy analysis revealed a fairly strong contribution of arm and shoulder muscles in the heel strike synergies (S1 and S4) and the switches between the modes of coordination were marked by a decrease in the involvement of arm muscles when the arm swing was in-phase with the leg swing. These phase-specific modulations could hence be directly related to the changes in kinematic behavior. Moreover, not all synergies were affected. Taken together, we rather support the notion of modular motor control, in which synergies can be modulated depending on the task while other synergies are robust across conditions (Nazarpour et al., 2012).

We used one-mode projections, commonly employed in bipartite networks (Murphy et al., 2018), of the muscle synergy weights to construct multiplex networks (Horvát and Zweig, 2012), with each layer reflecting a synergy. These synergy networks can reveal functional connections between multiple muscles in line with functional modules related to the biomechanical constraints of walking (Neptune et al., 2009). For example, next to the coordination-related coupling between contralateral arms and legs, we also found ipsilateral connections between arms and legs specific for the 2:1 locking mode. The networks of synergies S3 and S5 were dominated by activities important for push-off (GM) and foot raise (contralateral TA), but this modulation did not depend on the mode of coordination. When collapsing the multiplex network across layers, the synergy network only reflected the biomechanical characteristics of walking that kept the mechanisms underlying synergy formation opaque (Tresch and Jarc, 2009). Yet, the muscle synergy network approach supports the idea of functionally organized synergies that are modulated by changes in interlimb coordination.

The topology of the muscle synergy network showed clear similarities with the network derived from intermuscular coherence at lower frequencies (0.6–4 Hz, see Supplementary Material). Coherence at very low frequencies likely captures the co-variation of EMG envelopes which underpins the synergy analysis. Hence, both synergy and coherence networks may yield equivalent results, though, very low-frequency coherence might be difficult to estimate reliably due to the brevity of the gait cycles. At higher frequencies, the agreement between both types of networks was largely absent, as we did not observe a modular structure in the multiplex synergy network. This suggests that synergy and coherence analyses are complementary and potentially capture different aspects of motor control. As expected, the community structure of the coherence networks was closely related to the anatomical relationships of the muscles (Kerkman et al., 2018).

Higher frequency components of intermuscular coherence may indicate different functional pathways in the neuromuscular system, which were affected by the coordination between limbs. We found major changes in the 1:1 mode of coordination compared to the 2:1 mode and the transition, indicating a reorganization in the structure of common input during 1:1 coordination. The connectivity between 4 and 8 Hz was strongly increased between the arm and contralateral leg muscles in the 1:1 mode, indicative for altered afferent input (Bourguignon et al., 2019) and seemingly relevant for maintaining forward propulsion (cf. above). Connectivity in the frequency range of 8–22 Hz covers both alpha and low beta frequency ranges and have frequently been observed in intermuscular (Boonstra et al., 2015; Kerkman et al., 2018) and corticomuscular coherence (Conway et al., 1995; Boonstra et al., 2009b; Petersen et al., 2012; de Vries et al., 2016; Roeder et al., 2018). Although corticomuscular connectivity was not assessed in our study, we are tempted to interpret these frequency ranges as different neural pathways, possibly reflecting afferent and efferent inputs to spinal motor neurons, respectively (McAuley and Marsden, 2000; Rathelot and Strick, 2009; Bourguignon et al., 2019). The connectivity at 8–22 Hz was only affected when the legs and arms were in antiphase, i.e., in the 1:1 mode of coordination, with stronger long-distance connections between both lower back and leg muscles. First and foremost, the overall connectivity changed instead of a reorganization in connectivity patterns. That is, the conjunction between the upper and lower body muscles gained importance arguably because of an increasing demand of upper relative to lower body movements when walking faster. Finally, the connectivity in the frequency component of 22–60 Hz was less affected by changes in interlimb coordination.

The absence of neural connectivity during the 2:1 mode of coordination is in contrast to the kinematic coupling between the limbs. The increase in long-distance connectivity between the upper and lower limbs when switching to 1:1 coordination may indicate additional demands when switching to antiphase coordination. The absence of interlimb coupling in the EMG envelopes might indicate a largely passive contribution of the arm swing at slow walking speeds, while at higher speeds muscle activity is needed to actively establish interlimb coordination and possibly reduce the cost of walking (Collins et al., 2009). The active contribution of arm muscle activity in the 1:1 mode of coordination seemingly underlies the reorganization of muscle synergies. In our study, this reorganization was associated with increased functional connectivity between the arms and legs specifically at 4–22 Hz, which again implies increased common input to both arm and leg muscles (Boonstra et al., 2016). Muscle networks showed an abrupt change in network topology with increased long-distance connections when switching to a 1:1 mode of coordination. The increase in connectivity between arm and leg muscles is also reflected in the layers of synergy network corresponding to synergies S1 and S4, while muscle networks during quiet standing were mainly dominated by local connectivity (Boonstra et al., 2015; Kerkman et al., 2018). The switches in interlimb coordination were hence associated with distinct changes in the functional connectivity in the neuromuscular system reflecting common input to multiple muscles.

Admittedly, our results do not provide undeviating evidence for possible neural causes of synergy formation or stability of interlimb coordination. A promising future step could be to infer the dynamic coupling functions between muscle activation profiles that, in principle, do contain all information about the functional mechanisms underlying the interactions and prescribe the physical rule specifying how an interaction occurs (cf. Stankovski et al., 2017). We also have to admit that we did not directly assess the contribution of the supra-spinal inputs and it might be a “natural” step to evaluate these inputs using measures like partial directed coherence (e.g., Boonstra et al., 2015) or other directed information theoretic measures (e.g., Boonstra et al., 2019). While evidence about the functional role of intermuscular coherence is rapidly accumulating (Farmer et al., 1993; Boonstra et al., 2015, 2019; De Marchis et al., 2015), research on possible cortical contributions during whole-body movements comes with challenges (Gwin et al., 2010). Several studies already revealed the phasic modulation of corticomuscular coherence (Gwin et al., 2011; Gwin and Ferris, 2012; Roeder et al., 2018) and their importance of stabilizing modes of coordination (Bruijn et al., 2015). Interestingly, a recent experiment by Zandvoort et al. (2019) successfully identified cortical contributions to synergy formation by combining electroencephalography with EMG-based synergy analysis. Future work may adopt this approach to substantiate our suggestions about high-frequency, long-distant neural activation in the context of interlimb coordination and their sources in the central nervous system.

## Conclusion

The reorganization in muscle synergies and the concomitant alterations in coherence modulations of common neural input to multiple muscles highlight that switches in interlimb coordination are associated with changes in neuromuscular control. Network analysis of connectivity between all muscle pairs showed that the modularity of the neuromuscular system couples anatomical and functional linked muscles. The speed-induced transition to a 1:1 arm-leg frequency locking is accompanied by strong intermuscular coherence between upper and lower body muscles. This functional connectivity is particularly pronounced at higher frequencies indicating a significant long-distance neural interaction that accompanies the formation of muscle synergies.

## Data Availability Statement

The datasets generated for this study are available on request to the corresponding author.

## Ethics Statement

The studies involving human participants were reviewed and approved by Ethics Committee Human Movement Sciences of the Vrije Universiteit Amsterdam. The participants provided their written informed consent to participate in this study.

## Author Contributions

JK, AB, AD, and ND designed the experiment. JK and AB conducted the recordings and the analysis of the kinematics and the EMG. JK and TB performed the network analysis. All authors wrote the manuscript.

## Conflict of Interest

The authors declare that the research was conducted in the absence of any commercial or financial relationships that could be construed as a potential conflict of interest.

## Footnotes

1 In particular the increase in phase variability in the immediate vicinity of the behavioral switch in interlimb coordination resembles so-called critical fluctuations which implies the presence of a likewise critical slowing down, i.e., drastic increase of response time after (mechanical) perturbation.

2 As said, we investigated the dynamics in the vicinity of a phase transition.

3 Here, we would like to note that we verified that the amplitude normalization had little to no effect on the temporal and spatial representation of the muscle synergies.

4 Estimating muscle synergies per condition had only minor effects on both weightings and wave forms; details can be found in the Supplementary Material.
