# Supplementary material for: Muscle Synergies and Coherence Networks Reflect Different Modes of Coordination During Walking
Source: Front Physiol. 2020 Jul 24;11:751. doi: 10.3389/fphys.2020.00751 (PMC7394052; doi:10.3389/fphys.2020.00751)
Supplement: Supplementary file 1 [file Data_Sheet_1.pdf]

Muscle activity

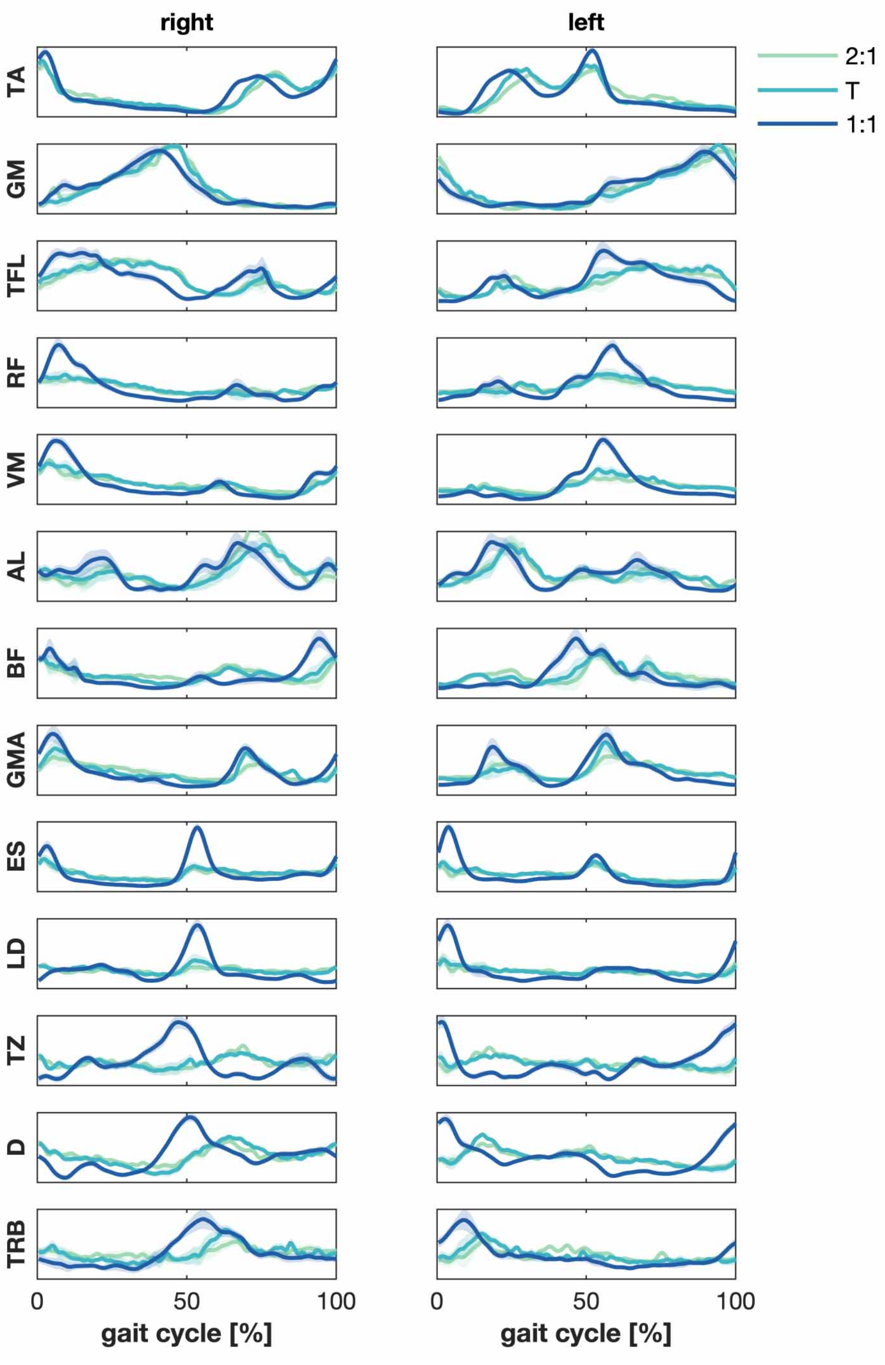

Figure S1. Grand average muscle activity patterns for all muscles in the different modes of coordination during the gait cycle. 0 and 100% indicate the right heel strike. Green, cyan and blue temporal patterns represent the 2:1, transition (T) and 1:1 mode of coordination, respectively. Error patches represent the standard error of the mean across subjects.

Muscle synergies per condition

We obtained both varying wave forms and weightings when muscle synergies were estimated per condition, i.e. the EMG data was not concatenated. Nevertheless, the wave forms and weightings were similar to the synergies estimated over the concatenated data.

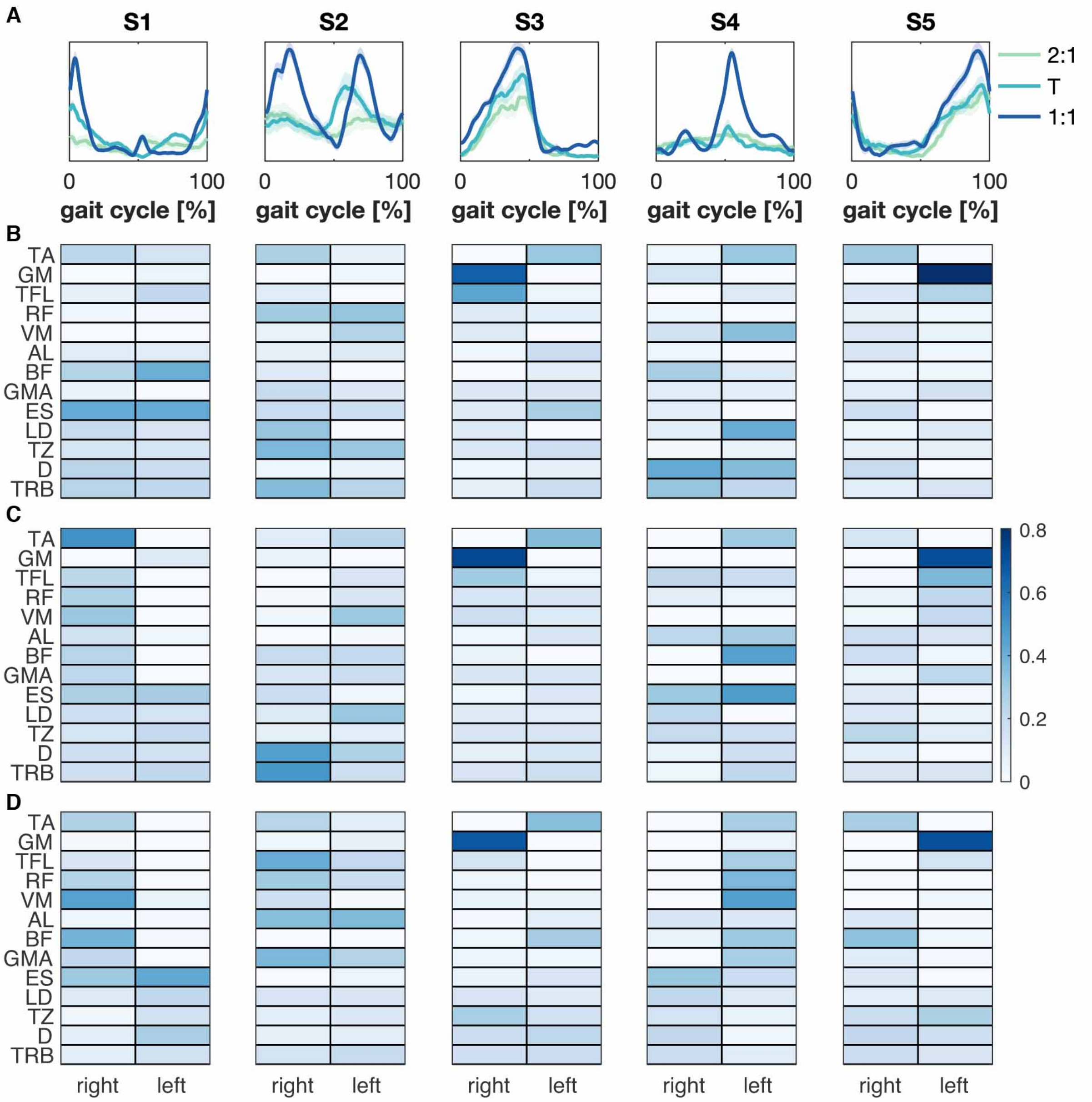

Figure S2. Muscle synergies estimated per mode of coordination. A) Muscle synergy wave forms. Green, cyan and blue represent the 2:1, transition (T) and 1:1 mode of coordination, respectively. **B)** Muscle synergy weightings of the 2:1, **C)** transition, **D)** 1:1 mode of coordination.

Comparison low frequency coherence and synergy networks

Muscle synergies reveal slow-temporal dynamics of muscle activity while intermuscular coherence is mainly focused on high-frequency connectivity between muscles. Both provide information about the function of the same neuromuscular system in different modes of coordination. Low-frequency intermuscular coherence is expected to display similarities with muscle synergies. To show this, we here discarded condition specific frequency information (e.g. stride time) by time normalisation of the stride. Subsequently, we estimated intermuscular coherence between all muscle pairs in the frequency range of 0.6-4 Hz in which 1 Hz represents the stride duration. We used the same procedure as described in the method section *Intermuscular coherence* but used a window of 5s instead of 200 ms, which allowed to focus on coherence at low frequencies with a frequency resolution of 0.2 Hz. We applied non-negative matrix factorisation over the coherence spectra and again used Eq. (1) to select the number of frequency components and estimated the community structure across frequency components and conditions. We examined the similarity of the community structure of the low and high frequency coherence networks by permutation testing (number of iterations = 10.000) of the Rand index and the adjusted Rand index. The Rand index is the sum of the edges present within the same and in different modules of both networks divided by the total number of edges in the networks. A Rand index of 1 implies that all edges are placed in the same module in both networks. The adjusted Rand index additionally accounts for grouping of the edges by chance (Fortunato, 2010; Qannari, Courcoux, & Faye, 2014).

Two modes were used to decompose the coherence spectra; $\lambda_c = 19\%$ and $\lambda_{1,2}^{(\mathrm{coh})} = [9, 10]\%$. One frequency component showed a peak at 1.5 Hz, while intermuscular coherence was high in the other frequency component at 2.5 and 3.5 Hz (Figure S3A). The community structure of the low-frequency coherence was very similar to the community structure of the coherence networks over the frequency range of 4-60 Hz: The Rand and adjusted Rand index were 0.85 and 0.63, $p < 0.001$, respectively. The legs were again mainly divided into two modules, though, the muscles at the medial and posterior side (AD and BF) of the right upper leg were part of the left leg module, and the trunk and arms formed one module. The coherence networks of 1:1 revealed the clearest similarities with the synergy networks (Figure S3B). The frequency component of 1.5 Hz showed high within leg connectivity and was very similar to the heel strike synergies (S1 and S4), while the connectivity in this network was also high within the trunk and arms. The latter was also shown in all synergies except of S2. Connectivity at 2.5 and 3.5 Hz was even stronger within and between the trunk and arms. This network also showed the high interlimb connectivity around the pelvis which was typical for S2. Other notable connectivity in the low-frequency coherence networks was found in the 2:1 condition with long-distance connectivity between the leg and the ipsilateral arm which was also shown S3 and S5 and related to the in-phase movement of the arm and leg.

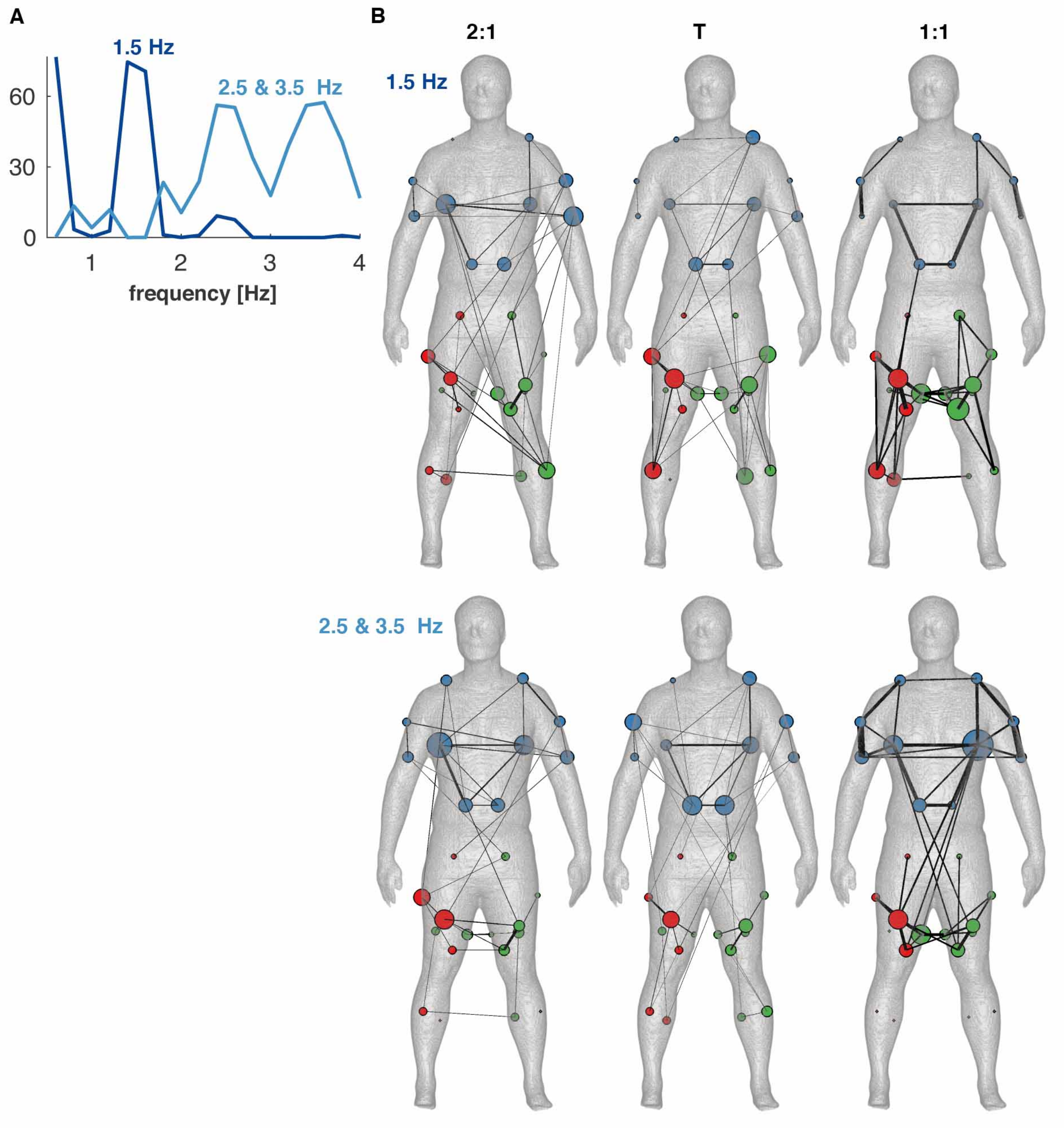

Figure S3. Minimally-connected multiplex coherence network for frequency range of 0-4 Hz. A) Frequency components and **B)** the corresponding coherence networks for the 2:1, transition (T) and 1:1 mode of coordination. The community structure is colour-coded and the node size and edge width represent degree and connectivity strength between muscles, respectively.

Community structure minimally-connected muscle networks

Thresholding of the edges is a common procedure in the analysis of networks. The removal of meaningful edges can induce ambiguities in the interpretation of differences between networks. Here we used a community structure algorithm to detect modules based on all the significant weighted edges in the networks. Another option to determine the community structure is to construct a minimally-connected binary network in which only the highest edges are considered for the determination of the clustering in the network (Didier, Brun, & Baudot, 2015).

We found that thresholding the synergy and coherence networks to construct a minimally-connected multiplex network barely affected the community structures of the networks (Figure S4). The synergy network consisted of two modules, one mainly at the lower legs, while the other one covered the pelvis and the upper body, but the division of the two modules does not seem to represent any clear anatomical or functional constraint and hence seemed not to be meaningful. The community structure of the coherence network, in contrast, was the same as the one of the unthresholded and weighted network and did resemble the anatomical and task constraints. Thresholding of the edges in this data set seemed not to affect the clustering of the network. The clustering in the network is probably driven by the edges with high connectivity.

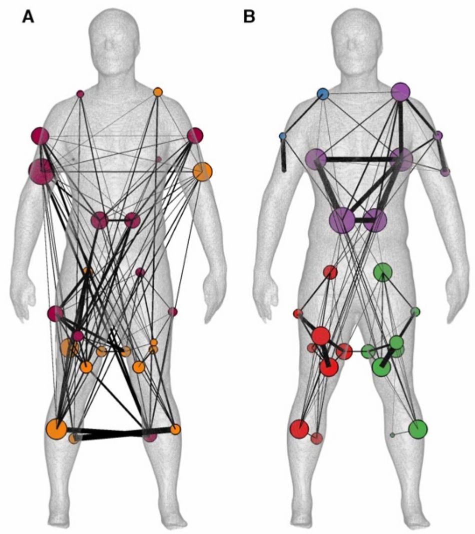

Figure S4. The community structure of the minimally-connected multiplex A) muscle synergy B) and coherence networks based on the synergy and coherence spectra muscle weightings. Community structure is visualised by colour-coded nodes and the average degree across layers of every muscle is displayed as node size on the body mesh (Makarov et al., 2015). The edge width is based on the average connectivity across layers between the muscles in either the synergy or coherence network.

References

Didier, G., Brun, C., & Baudot, A. (2015). Identifying communities from multiplex biological networks. *PeerJ*, *3*, e1525. https://doi.org/10.7717/peerj.1525

Fortunato, S. (2010). Community detection in graphs. *Physics Reports*, *486*(3–5), 75–174. https://doi.org/10.1016/j.physrep.2009.11.002

Qannari, E. M., Courcoux, P., & Faye, P. (2014). Significance test of the adjusted Rand index. Application to the free sorting task. *Food Quality and Preference*, *32*, 93–97. https://doi.org/10.1016/j.foodqual.2013.05.005
